# Effect of Nitrogen Atmosphere Annealing of Alloyed Powders on the Microstructure and Properties of ODS Ferritic Steels

**DOI:** 10.3390/ma17081743

**Published:** 2024-04-10

**Authors:** Agata Strojny-Nędza, Katarzyna Pietrzak, Iwona Jóźwik, Bartosz Bucholc, Edyta Wyszkowska, Łukasz Kurpaska, Agnieszka Grabias, Agnieszka Malinowska, Marcin Chmielewski

**Affiliations:** 1 Łukasiewicz Research Network Institute of Microelectronics and Photonics, 02-668 Warsaw, Poland; agata.strojny.nedza@imif.lukasiewicz.gov.pl (A.S.-N.); iwona.jozwik@imif.lukasiewicz.gov.pl (I.J.); bartosz.bucholc@imif.lukasiewicz.gov.pl (B.B.); agnieszka.grabias@imif.lukasiewicz.gov.pl (A.G.); agnieszka.malinowska@imif.lukasiewicz.gov.pl (A.M.); 2 Institute of Fundamental Technological Research, Polish Academy of Sciences, 02-106 Warsaw, Poland; kpietrzak@ippt.pan.pl; 3 NOMATEN Centre for Excellence, National Centre for Nuclear Research, 05-400 Otwock, Poland; edyta.wyszkowska@ncbj.gov.pl (E.W.); lukasz.kurpaska@ncbj.gov.pl (Ł.K.)

**Keywords:** spark plasma sintering, ODS ferritic steel, mechanical alloying, Mössbauer spectroscopy, nanoindentation

## Abstract

Oxide Dispersion Strengthened (ODS) ferritic steels are promising materials for the nuclear power sector. This paper presents the results of a study on the sintering process using the Spark Plasma Sintering (SPS) technique, focusing on ODS ferritic steel powders with different contents (0.3 and 0.6 vol.%) of Y_2_O_3_. The novelty lies in the analysis of the effect of pre-annealing treatment on powders previously prepared by mechanical alloying on the microstructure, mechanical, and thermal properties of the sinters. Using the SPS method, it was possible to obtain well-densified sinters with a relative density above 98%. Pre-annealing the powders resulted in an increase in the relative density of the sinters and a slight increase in their thermal conductivity. The use of low electron energies during SEM analysis allowed for a fairly good visualization of the reinforcing oxides uniformly dispersed in the matrix. Analysis of the Mössbauer spectroscopy results revealed that pre-annealing induces local atomic rearrangements within the solid solution. In addition, there was an additional spectral component, indicating the formation of a Cr-based paramagnetic phase. The ODS material with a higher Y_2_O_3_ content showed increased Vickers hardness values, as well as increased Young’s modulus and nanohardness, as determined by nanoindentation tests.

## 1. Introduction

One of the most important research directions in nuclear history is the development of steel-based energy conversion materials for future fusion or enhanced fission reactors [1,2,3,4]. The extremely harsh operating conditions prevailing within these reactors are due to the accumulation of simultaneous factors, such as dynamic thermal loads combined with aggressive chemical coolants and intense radiation fluxes [5].

Much effort has been devoted worldwide to improving known and available materials and developing new ones to withstand these conditions and thus contribute to advancements in the energy sector. Oxide dispersion-strengthened (ODS) alloys, particularly ferritic steels, are the most studied group of materials. They are promising candidates for fuel cladding in next-generation fission reactors, as well as first wall and cladding materials for future fusion reactors operating at 750 °C or higher, due to their good oxidation and corrosion resistance and perfect tolerance for high-energy neutron fluxes [6,7,8]. They also exhibit excellent swelling and creep resistance at elevated temperatures [9]. Although these materials have all these advantages, they may not be sufficient under critical conditions involving dynamic thermal loads combined with aggressive chemical and radiation environments. For this reason, new material solutions are still being sought worldwide by modifying composition, techniques of manufacturing, or methods of powder preparation. Among the most popular is the ferritic steel developed at Oak Ridge National Laboratory—14YWT, with a nominal composition (in wt.%) of Fe-14%Cr-3%W-0.4%Ti and a 0.3% addition of Y_2_O_3_ nanoparticles [10]. It is believed that the presence of finely dispersed oxide particles increases the strength of the alloy by pinning dislocations, thus hindering their movement. In addition, they also reduce the mobility of grain boundaries, thereby improving the high-temperature creep resistance of the material. The most commonly used oxides for strengthening ferritic steel are nanoparticles of yttrium oxide, which is one of the most thermodynamically stable compounds. Y_2_O_3_ particles serve as a strengthening phase due to their high chemical stability, high melting point, and low solubility in the metal matrix. Therefore, the strengthening effect resulting from uniformly distributed oxides is effective over a wide temperature range. The interfaces between the matrix and the ceramic nanoparticles provide high internal surface energy, which allows for the annihilation of point defects, thanks to which undesirable effects such as material swelling are observed only at much higher radiation doses [11,12]. According to the literature, ODS materials are usually obtained through powder metallurgical routes, including mechanical alloying (MA) followed by consolidation by hot isostatic pressing (HIP) and hot extrusion (HE) [13,14,15]. In recent years, however, there has been a marked increase in interest in the use of alternative methods such as spark plasma sintering (SPS) [16,17,18,19]. SPS (also known as the Field-Assisted Sintering Technique) is a process in which powder samples are loaded into a conductive die (usually graphite) and then consolidated by the application of uniaxial pressure and the passage of electric current. A key feature of the SPS technique is its ability to achieve high heating and cooling rates [20]. As a result, the annealing time of densified materials can be significantly reduced, ultimately resulting in limited grain growth. As is well known, grain growth is an undesirable phenomenon that ultimately reduces the hardness of the material (according to the Hall–Petch equation) and therefore increases the likelihood of elastic fracture when the specimen is subjected to stress [21,22]. In addition, other advantages of SPS over HE and HIP include rapid processing, ease of use, and the ability to control each stage of the sintering process. The promising results of the work of Boulnat et al. stimulated further research using a combination of MA and SPS techniques [22]. One of the first comprehensive studies on the production of ODS Eurofer (Fe-9%Cr-1%W-0.2%V-0.1%Ta-0.3%Y_2_O_3_) using MA and SPS was published by Fu et al. [23]. They used different combinations of MA and SPS parameters to optimize the fabrication process. The fabricated material exhibited a bimodal microstructure with homogeneously dispersed nanoscale Y_2_O_3_, which was found to be beneficial for mechanical properties. Their work emphasized the influence of coarse carbides decorating grain boundaries, resulting from the diffusion of carbon atoms from the graphite matrix, on the mechanical properties.

The SPS technique was also used by Macía et al. to improve the efficiency of ODS steel production [24]. After sintering at 1100 ℃, they obtained high-density (up to 99.8%) ferritic steels with a composition of Fe-14%Cr-5%Al-3%W-0.4%Ti-0.25%Y_2_O_3_-0.6%Zr. The material exhibited promising mechanical properties in the higher temperature range, which was attributed to microstructural refinement (resulting from the application of field-assisted sintering techniques and the addition of Zr).

A major concern in the production of ODS materials is the need for strict control of the chemical composition at each stage of the process. Some intermediate steps can be implemented to reduce, for example, the number of metal oxides formed on the surface as a result of contact with the ambient atmosphere, which may be unavoidable in some production processes.

This paper presents the results of our studies on the sintering of ODS ferritic steel powders with different (0.3 and 0.6 vol.%) Y_2_O_3_ contents using MA and SPS techniques. The novelty presented in this work lies in the analysis of the effect of pre-annealing treatment of powders prepared by MA on the microstructure, mechanical, and thermal properties of the sintered material. Comprehensive studies, including techniques such as scanning electron microscopy (SEM), X-ray diffraction (XRD), Mössbauer spectroscopy, laser flash analysis (LFA), Vickers hardness, and nanoindentation, have been carried out to characterize the microstructure and some selected properties of the obtained ODS sinters.

## 2. Materials and Methods

The ODS steel powder blends were prepared by MA of Fe, Cr, W, Ti, and two amounts of Y_2_O_3_ 0.3% and 0.6% by volume. The composition of the powders intended for the matrix (wt.% Fe-14%Cr-2%W-0.3%Ti) was chosen based on an analysis of the state of the problem and our own previous work [8]. The amount of ceramic phase was chosen based on literature analysis.

The metallic powders of Fe, Cr, W, and Ti are commercially available materials provided by the AlfaAesar company (Haverhill, MA, USA) with purities of: 99.5, 99, 99.9, and 99.5%, respectively. The yttria ceramic powder was prepared in our laboratory using the precipitation method. The particle size distribution of the initial powders was studied using the CLEMEX television image analysis system. As a result of calculations based on Feret functions, the average Feret diameter (d) was obtained.

Mechanical alloying of the starting powders was performed in a planetary ball mill (Pulverisette 6, Fritsch, Pittsboro, NC, USA) at an effective speed of 350 rpm, using a vial (250 mL) and balls (Ø = 10 mm) both made of steel. In each case, a ball-to-powder-weight ratio of 10:1, a total time of 20 h, and a high purity argon atmosphere were used. The choice of grinding time of 20 h was a result of our previous work, which can be found in article [8]. In order to study the correlation between the properties of resulting sinters from annealed and unannealed powders, a part of the powder was subjected to an annealing process (AP) at 600 °C under a nitrogen atmosphere, and the remaining part was the reference material.

After grinding, the obtained ODS powders with two different amounts of yttrium oxide were sintered using an SPS apparatus (own design). The densification process was performed in a graphite die (diameter 10 mm) within a vacuum chamber at a sintering temperature of 1075 °C, a heating rate of 10 °C/min, a holding time of 5 min, and a pressure of 50 MPa. The parameters of the sintering process were chosen on the basis of previous experimental work [8]. As a result, cylindrical samples with a diameter of 10 mm and a height of 3 mm were obtained. Qualitative phase analysis of powder materials after milling was performed using the X-ray powder diffraction (XRD) method. Measurements were performed with a Rigaku SmartLab 3kW universal X-ray diffractometer (Rigaku, Tokyo, Japan) equipped with a Cu X-ray tube and a high-speed 1D silicon semiconductor strip detector (D/teXUltra 250). The powder diffraction patterns were measured in the reflection Bragg–Brentano geometry (θ/2θ scan) using the continuous scan mode. Qualitative and quantitative XRD phase analyses, including refinement of structural parameters with the Rietveld method as well as estimation of average crystallite sizes with the Williamson–Hall method, were performed using a PDF-4+ 2023 database and PDXL2 software (Version 2.8.3.0) supplied by Rigaku. For the samples of ODS steel powders obtained by MA, ^57^Fe Mössbauer spectroscopy measurements (own design) were performed at room temperature in the transmission geometry in order to study the changes in local atomic arrangements around Fe nuclei induced by annealing.

The relative density of the sintered samples was measured using the Archimedes method according to [25] standard ASTM:B962-08. Samples of ODS steels were characterized by high resolution SEM using two systems: the Carl Zeiss Auriga CrossBeam Workstation and the Hitachi SU8230 (Warsaw, Poland). Both systems, equipped with sophisticated advanced detection systems, allowed for the application of primary energy within a wide range, i.e., 0.5–10 keV. Elemental analysis of precipitates and bulk material was performed using energy dispersive X-ray spectroscopy (EDS) (Bruker, Billerica, MA, USA). Samples for microstructural investigations were embedded in epoxy resin and polished to a high gloss.

The thermal conductivity of ODS alloys was measured in the temperature range of 50–600 °C using the Laser Flash Analyzer LFA457 (Netzsch, Selb, Germany) under an argon atmosphere for samples with a diameter of 10 mm and a length of 2 mm. The surfaces of the samples were covered with a thin layer of graphite. The whole set of experimental data was fitted by applying the Cape–Lehman theoretical model with pulse correction, which takes into account radiative heat loss. The thermal diffusivity (a) and, in most cases, the specific heat (c_p_) can be obtained from the measured signal. If the density (ρ) and specific heat are known, the thermal conductivity (λ) can be determined from the relationship:

Λ = c_p_·a·ρ (1)

The measurements of the specific heat (used in the calculation of thermal conductivity) of the obtained samples were carried out with a STA 449 Jupiter F5 (Netzsch, Selb, Germany) fine thermal analyzer—using the Differential Scanning Calorimetric (DSC) method with heat flow. Measurements were performed on 4.0 mm × 4.0 mm × 1.0 mm samples in the temperature range 50–600 °C.

The coefficient of linear thermal expansion (CTE) of the materials was measured on the DIL 402 Expedis Select (Netzsch, Selb, Germany) in the temperature range of 50–600 °C under a protective atmosphere of argon.

Hardness (HV0.1) was tested with a Durascan 10 (Struers, Emcotests, Champigny-sur-Marne, France) using a Vickers diamond indenter with a load of 0.981 N applied for 10 s. The hardness results were averaged over three indentations per specimen.

**Figure 1:**
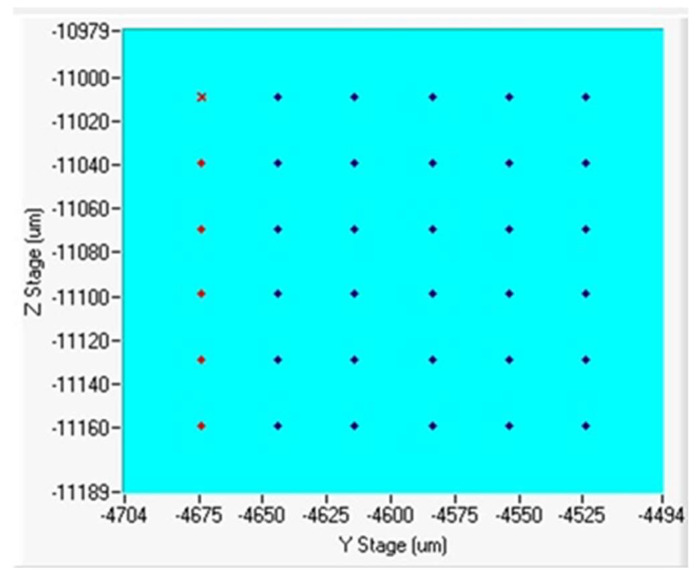
Illustrated scheme of the mapping during nanoindentation testing.

Nanomechanical properties such as Young’s modulus and nanohardness were measured by nanoindentation using the NanoTest Vantage system from Micro Materials Ltd. (Wrexham City, UK). A Synton-MDP diamond Berkovich indenter was used. Prior to the experiment, the instrument was calibrated, and the Diamond Area Function (DAF) of the indenter tip was determined. This was done by performing a series of indentations at different loads on fused silica (standard material). The DAFs were calculated for each load and used at each stage of the results analysis. In this work, 36 indentations were made with a load of 50 mN and a spacing of 30 µm between indentations to obtain the hardness and Young’s modulus distribution on a surface of 150 μm × 150 μm (about 0.0225 mm^2^). Nanomechanical measurements were performed to evaluate the mechanical properties of the two compositions presented. Figure 1 shows the indentation grids obtained for ODS 0.3% Y_2_O_3_ and 0.6% Y_2_O_3_. Depending on the % of Y_2_O_3_ phase addition, the applied load corresponded to an indentation depth of 540–657 nm. To eliminate the creep of the sample, the maximum load was held for 1 s, and the dwell time for drift correction was 60 s. Loading and unloading curves were 10 and 5 s, respectively.

Then, the hardness was extracted from the indentation load–displacement curves during one cycle of loading and unloading, based on the Oliver–Pharr method [26]. The calculation of nanohardness was facilitated by the following relationship:

H = P_max_/A, (2)

where P_max_ represents the maximum load, and A is the projected contact area at a given peak load. In addition, Youngs’s modulus was calculated using the following equation:

(3) $$\frac{1}{E_{r}}=\frac{1-\upsilon^{2}}{E}+\frac{1-\upsilon_{i}^{2}}{E_{i}},$$

where *E* and *υ* are the Young’s modulus and Poisson’s ratio of the specimen, respectively, and *E_i_*, *υ_i_* are the Young’s modulus and Poisson’s ratio of the indenter, and *E_r_* is the reduced Young’s modulus calculated by the system.

## 3. Results and Discussion

### 3.1. Powders and Sinters Characterization

**Figure 2:**
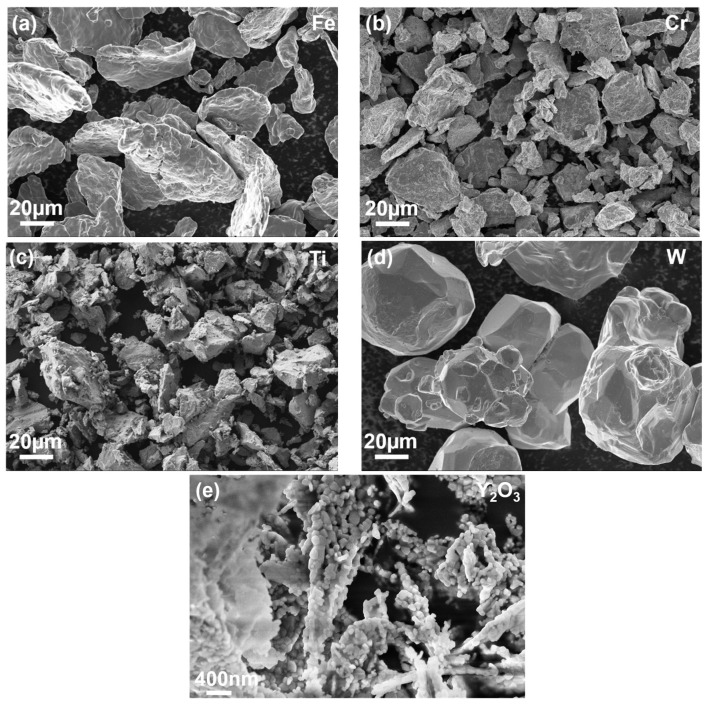
SEM micrographs of initial powders; (**a**) Fe, (**b**) Cr, (**c**) Ti, (**d**) W, and (**e**) Y_2_O_3_.

The average particle size of the powders Fe, Cr, W, and Ti were determined to be d(Fe) = 30 µm, d(Cr) = 6 µm, d(W) = 62 µm, and d(Ti) = 27 µm, respectively. In the case of Y_2_O_3_ powder, the dimension d(Y_2_O_3_) = 70 nm represents the average size of the crystallites. The microstructure of the starting powders is shown in Figure 2.

**Figure 3:**
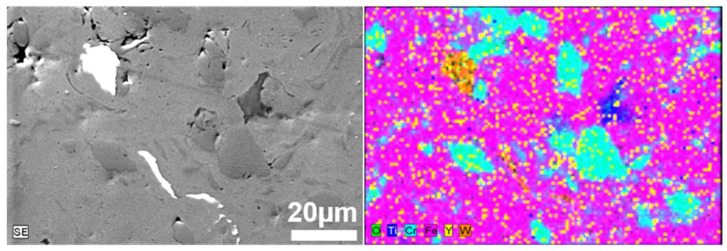
SEM/EDS micrographs of the cross-section of Fe-14Cr-2W-0.3Ti-0.3Y_2_O_3_ powder mixture obtained after 1 h of milling.

**Figure 4:**
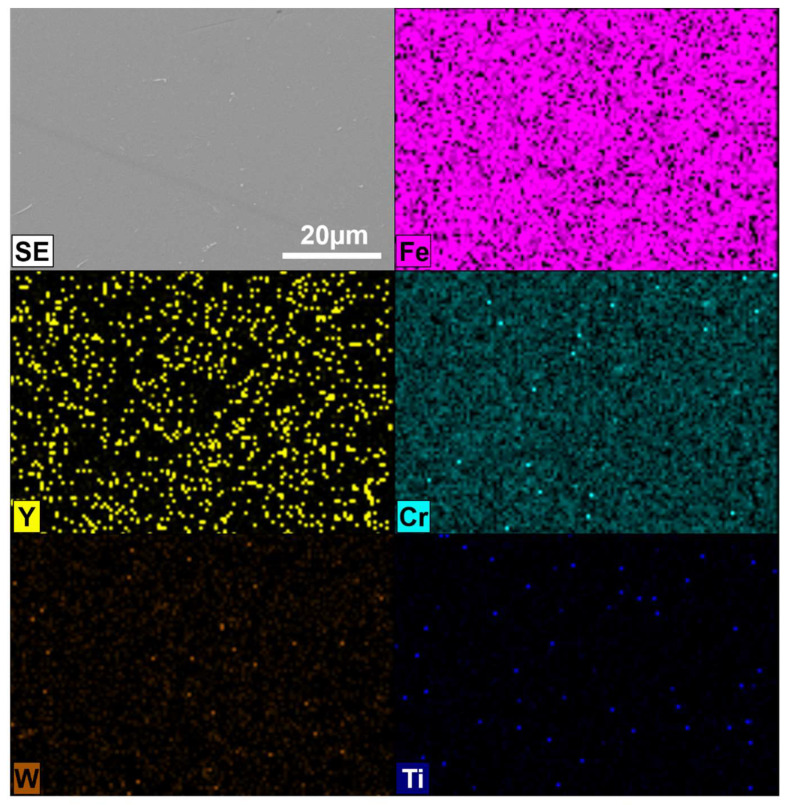
SEM/EDS micrographs of the cross-section of Fe-14Cr-2W-0.3Ti-0.3Y_2_O_3_ powder mixture obtained after 20 h of milling.

For better visualization of the powder mixture, metallographic cross-sections in polymer resin were prepared. The results of SEM/EDS images of prepared powders after 1 h and 20 h of milling are shown in Figure 3 and Figure 4, respectively.

**Figure 5:**
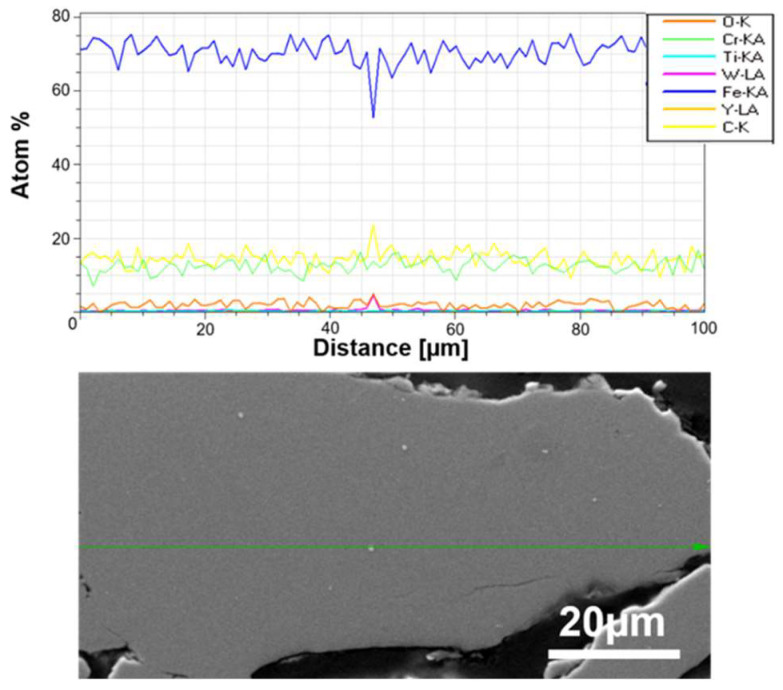
SEM micrographs of the cross-sections of ODS mixtures after 20 h of milling powders and linear element distribution along the marked green arrow in Fe-14Cr-2W-0.3Ti-0.3Y_2_O_3_.

An example of SEM observations with linear EDS analysis of prepared powders is presented in Figure 5.

The size of the individual grains decreases with grinding time, and phase contrast shows the uniformity of the mechanically alloyed structures. The only exception is WC, which is visible as small individual grains in the alloy (Figure 5). EDS analysis confirmed the homogeneous (sub-micron) distribution of the constituents of the individual fragments of the mixture. It was found that 20 h was a sufficient grinding time, after which the mixture obtained had a submicron structure with an evenly distributed disperse phase (Y_2_O_3_) (Figure 4). For a Y_2_O_3_ content of 0.6% by volume, a similarly uniform distribution of components was found.

**Figure 6:**
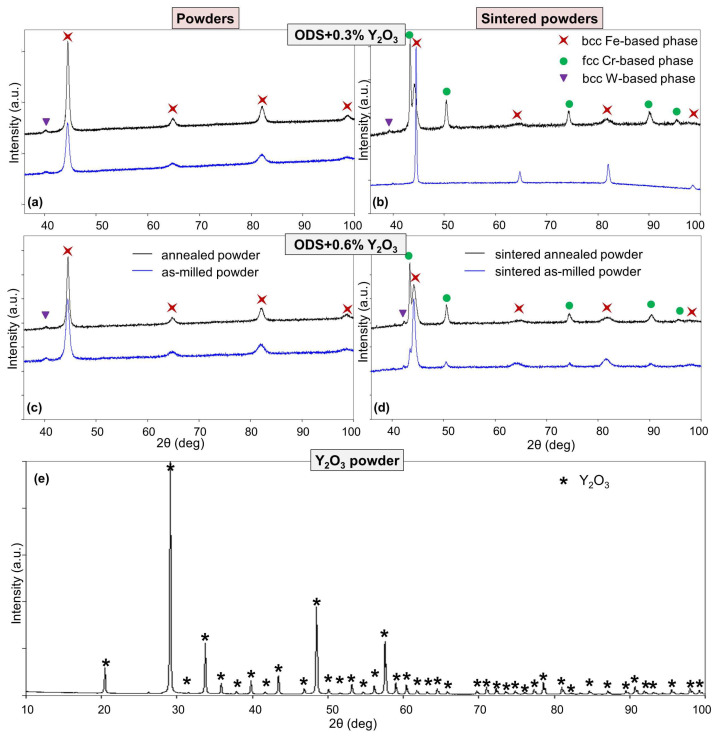
X-ray diffraction results: (**a**) as-milled and annealed ODS-0.3% Y_2_O_3_ powders, (**b**) sintered ODS-0.3% Y_2_O_3_ powders, (**c**) as-milled and annealed ODS-0.6% Y_2_O_3_ powders, (**d**) sintered ODS-0.6% Y_2_O_3_ powders, (**e**) Y_2_O_3_ powder.

The results of qualitative XRD analysis for the ODS-0.3% Y_2_O_3_ and ODS-0.6% Y_2_O_3_ annealed and unannealed powders and sinters are shown in Figure 6a–d. For all powder mixtures after MA, the basic bcc phase of the α-Fe (ferritic) type with the Im-3m (229) space group (i.e., Fe-based solid solution with the addition of Cr and other dopants) and most probably the separation of the trace amount of the bcc W-based phase with the same space group were observed (Figure 6a,c). In the case of annealed powders, the X-ray diffraction lines associated with the Fe-based phase become narrower and more intense, indicating some kind of ordering of the crystal structure that results in an increase in coherent scattering regions. In addition, a slight increase in the average crystallite size from about 7 nm to about 13 nm was observed. The sintering process results in the formation of a paramagnetic fcc Cr-based phase (Fm-3m (225) space group), most likely with some iron content. The sintered as-milled ODS-0.3% Y_2_O_3_ sample is an exception. The relative intensity of the diffraction lines corresponding to the fcc Cr-based phase is greater in the pre-annealed samples, indicating an increase in the content of this phase in the samples at the expense of the ferritic phase. A strong broadening of the diffraction lines associated with the Fe-based phase indicates some kind of disordering of its structure that results in a decrease in coherent scattering regions. The diffraction lines associated with the Fe-based phase (except for the sintered as-milled ODS-0.3% Y_2_O_3_ case) and the weak lines of the W-based structure in sintered materials are shifted compared to powder samples. This indicates a slight increase in lattice parameters in the Fe-based phases and a decrease in lattice parameters in the W-based phases, most probably caused by variations in the stoichiometry of the solid solutions.

Additional XRD analysis of the Y_2_O_3_ powder added during the ODS preparation proved that it was a single-phase crystalline material (Figure 6e). The Y_2_O_3_ diffraction lines were not detected in the XRD patterns in Figure 6a–d because the sensitivity of the method is limited to the order of tenths of a weight percent.

**Figure 7:**
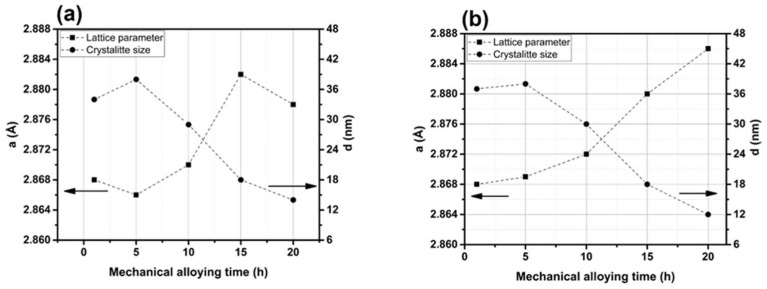
Lattice constant and crystallite size based on the example of mixtures containing: (**a**) 0.3% Y_2_O_3_ and (**b**) 0.6% Y_2_O_3_.

Figure 7 shows the basic parameters of the powder, such as lattice constant (a) and average crystallite size (d), plotted as a function of MA time for a mixture containing 0.3% and 0.6% Y_2_O_3_. These parameters were determined from data obtained by the XRD technique. The lattice constant, a, refers to a ferritic phase with a body-centered cubic crystal structure. After longer periods of effective grinding (15 and 20 h), a pronounced increase in the value of the lattice constant was observed. Similar behavior was observed for the 0.6% Y_2_O_3_ content. This effect is well known in MA processes and is attributed to the dissolution of alloying elements in the crystal lattice of the matrix (here Fe) during milling and to phenomena related to severe plastic deformation [27]. Based on the analysis of the crystallite size, longer milling times resulted in significant refinement of the crystalline structure of the material. After an initial slight increase between the first and fifth hour of the process, there was a significant decrease in the parameter d—from 38 to 14 nm after 20 h. The refinement of the crystal structure results from an extreme increase in defect densities (especially dislocations) [28], which occur when the powder particles collide with the grinding balls and the walls of the container. The estimated crystallite sizes are similar to those reported by Nowik et al. [27].

**Figure 8:**
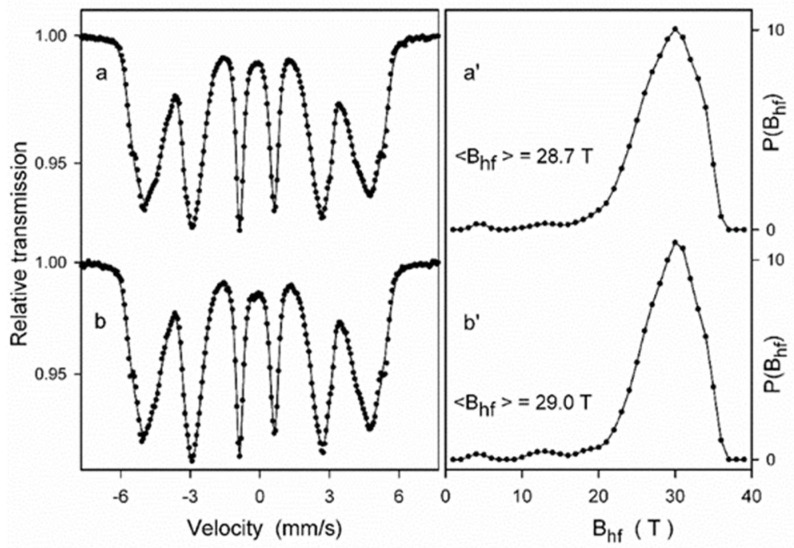
Mössbauer spectra of the as-milled (**a**) and annealed (**b**) ODS-0.3% Y_2_O_3_ powder samples and the corresponding hyperfine field distributions P(B_hf_) (**a’**,**b’**) for (**a**,**b**) powders mixtures, respectively.

Mössbauer spectroscopy measurements were performed for the ODS steel powder mixtures with different contents of Y_2_O_3_ (0.3% and 0.6%) directly after the milling process as well as after annealing. Figure 8 presents the Mössbauer spectra of the ODS-0.3% Y_2_O_3_ sample fitted with the histogram method of the distribution of magnetic hyperfine fields P(B_hf_). Fairly symmetric and broad peaks observed in Figure 8a’,b’ in the range of hyperfine fields from about 20 to 35 T indicate the formation of a solid solution based on α-Fe. The shape of the P(B_hf_) distribution changes slightly after powder annealing. A visible decrease in the distribution width is accompanied by a small increase in the average hyperfine field <B_hf_>. These features evidence subtle changes in the atomic arrangement around Fe nuclei induced by annealing, which may be attributed to the increase of local atomic order in the solid solution. Similar differences between the as-milled and annealed powders were observed for the ODS-0.6% Y_2_O_3_ sample.

**Figure 9:**
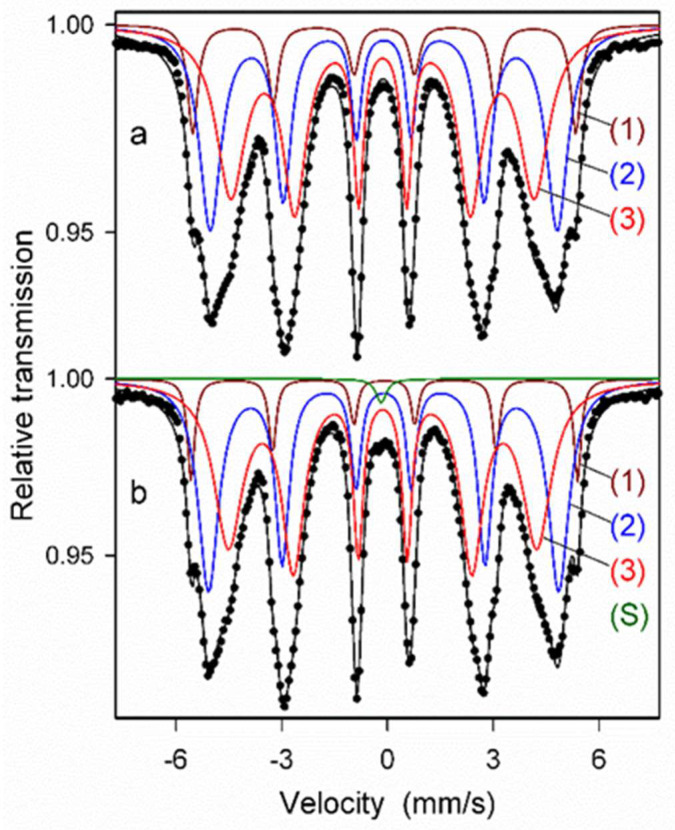
Mössbauer spectra of as-milled (**a**) and annealed (**b**) ODS-0.3% Y_2_O_3_ powder samples with discrete components: (1–3)—sextets associated with bcc Fe-based phases, (S)—a single line associated with a Cr-based phase.

**Figure 10:**
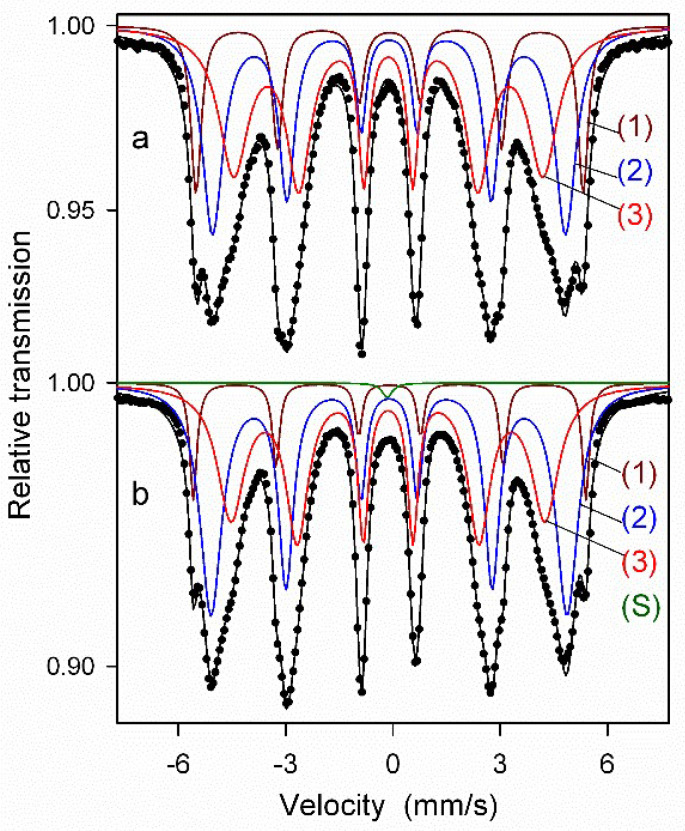
Mössbauer spectra of as-milled (**a**) and annealed (**b**) ODS-0.6% Y_2_O_3_ powder samples fitted with discrete components: (1–3)—sextets associated with bcc Fe-based phases, (S)—a single line associated with a Cr-based phase.

Next, the Mössbauer spectra were fitted with several discrete components that allowed for qualitative and quantitative analysis of the structure of the ODS samples (Figure 9 and Figure 10, Table 1). All spectra were fitted with three magnetic sextets, as suggested by the non-smooth shape of the main peak in the P(B_hf_) distributions in Figure 8. These sextets are typical for ferritic steel with a Cr content of 14 at.% [29,30]. The sextets show different values of hyperfine fields and spectral fractions, originating mainly from the distribution of Cr content in the ferritic phase. In general, the addition of Cr to the α-Fe-based phase causes a decrease in its hyperfine field. For both as-milled powders (Figure 9a and Figure 10a), the magnetic spectral components are assigned according to their hyperfine fields as follows: (1) B_hf1_ = 33.6 T—α-Fe-based phase with no Cr atoms as nearest neighbors, (2) B_hf2_ = 30.6 T, and (3) B_hf3_ = 26.7 T—solid solutions of Cr in the ferrite phase with a gradual increase of Cr atoms in Fe environments. A visible increase in the hyperfine field values of all sextets by about 0.3–0.5 T is observed after annealing (Figure 9b and Figure 10b) as in the case of the P(B_hf_) distributions. A significant decrease in the relative fraction of the sextet (1) seen after annealing of both samples indicates further distribution of Cr atoms in the solid solution. Furthermore, an additional spectral component (a single line) is revealed in the Mössbauer spectra of the annealed samples, with a small spectral fraction of about 1%, which is attributed to the formation of a paramagnetic Cr-based phase with some iron content [29,30]. It should be noted that Mössbauer spectroscopy is a very sensitive tool capable of detecting even small amounts of iron-containing phases. Thus, the Mössbauer results indicate an early formation of the Cr-based phase, which is already evident in the pre-annealed samples. This phase is clearly visible in the XRD patterns obtained for the sintered powders (Figure 6).

### 3.2. Characterization of ODS Sinters

Table 2 shows the results of measurements of physical and thermal properties of the obtained ODS sinters. The density of the obtained ODS sinters was tested using the Archimedes method. The values obtained for density, thermal conductivity, specific heat, and coefficient of linear expansion are shown in Table 2. The abbreviation AP indicates that the sample was sintered from a previously annealed powder.

**Figure 11:**
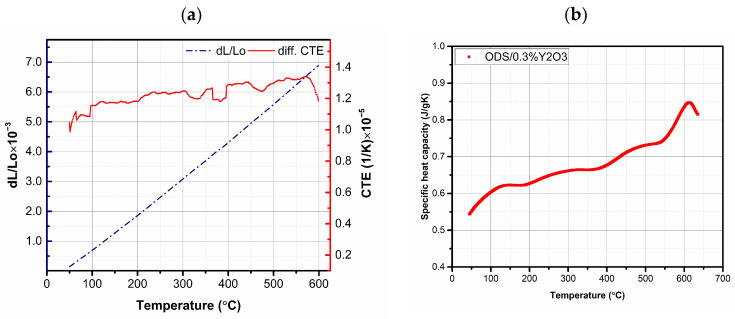
Relationship between the coefficient of linear expansion (CTE) and relative elongation (**a**) and specific heat (**b**) as a function of temperature for the ODS + 0.3% Y_2_O_3_ sample.

It was found that, in all the cases studied, the application of the SPS method allowed for the obtainment of well-compacted materials with a density higher than 98% of the theoretical one. Higher densities were observed for sinters in which powders were used after the annealing process (AP). These results were consistent with microstructure studies. When analyzing the obtained values of the relative density of the sinter, it should be considered that (i) the theoretical density (as in most publications) was calculated from the rule of mixtures on the basis of the densities of individual elements, and (ii) the presence of stoichiometric and non-stoichiometric compounds of oxides and carbides (with much lower density) formed during the manufacturing process (MA + SPS) and resulting from the conditions of the processes (e.g., graphite matrix) were not taken into account. In the next step, the thermal properties of the obtained materials were tested. Due to the insignificant number of literature reports on the study of thermal properties of ODS ferritic steels, thermal characterization of the obtained materials was performed. The values of specific heat and coefficient of linear expansion in the temperature range 50–600 °C were measured (Table 2 shows the values obtained at 50 °C for all tested materials). In terms of Cp and CTE, all samples showed very similar behavior; example curves of Cp and CTE values as a function of temperature are shown in Figure 11.

**Figure 12:**
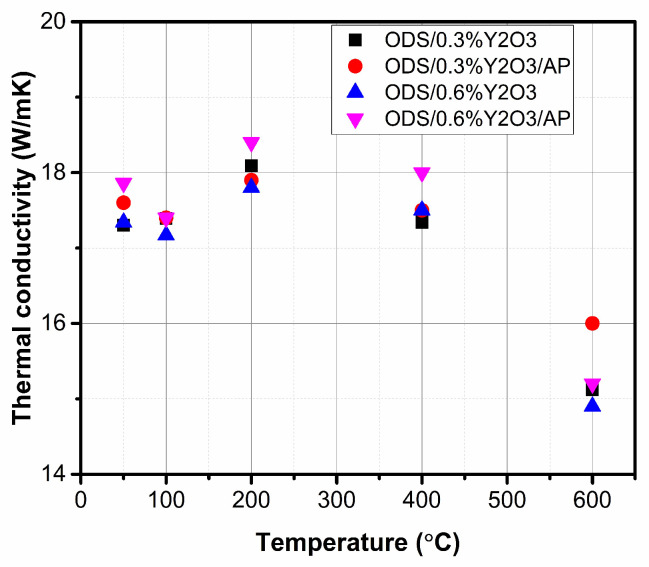
Thermal conductivity in the temperature range for obtained materials with different amounts of Y_2_O_3_.

The obtained values of specific heat and coefficient of linear expansion were used in thermal diffusivity measurements to evaluate the thermal conductivity. The obtained values of thermal conductivity of ODS materials with the addition of 0.3 and 0.6% yttrium oxide as a function of temperature are shown in Figure 12.

The obtained values of thermal conductivity are at a similar level for all tested materials. The sinters were characterized by high density, indicating negligible porosity in the structure, which usually reduces the value of thermal conductivity. Submicron precipitates of oxide and/or carbide type, observed both at grain boundaries and within the grain region, did not significantly affect the thermal conductivity of sintered as-milled and annealed powders.

**Figure 13:**
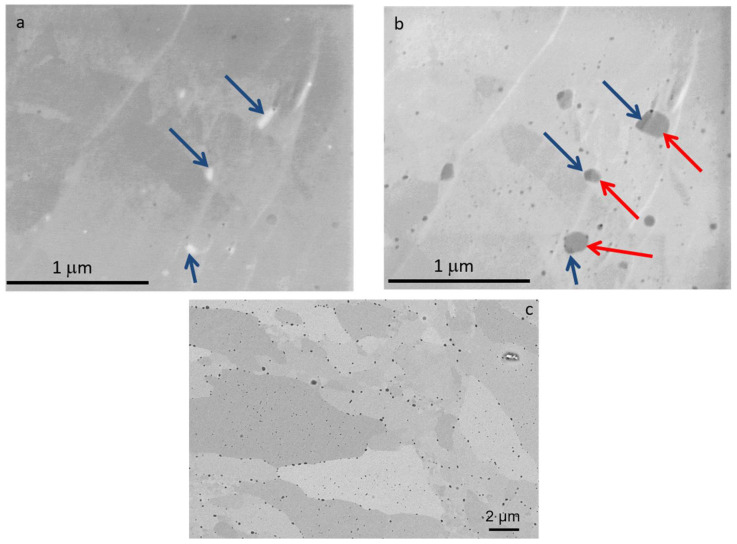
SEM micrographs of the surface of ODS steel sample Fe14Cr2W0.3Ti + 0.3% Y_2_O_3_ (ODS 0.3% Y_2_O_3_): (**a**) SE1 image and (**b**) BSE image, both collected at 1 keV primary electron beam energy; blue arrows indicate highly resistive/low atomic number precipitates; red arrows—higher atomic number precipitates; (**c**) BSE images collected at 10 keV.

The microstructure of the obtained sinters was analyzed. Different values of primary beam energies were applied with the aim of using different detectors to identify the types of precipitates [8]. Typical SEM micrographs obtained using backscattered electrons (BSEs) for the studied ODS alloys at 10 keV are shown in Figure 13. The analysis of the structures of the obtained ODS materials with different contents of yttrium oxide confirmed that the application of the SPS technique obtained materials with a high degree of densification without any defects, i.e., with a limited amount of pores and without visible microcracks and structural discontinuities. During the consolidation process, oxide precipitates of different compositions form within the matrix—this is confirmed by SEM images obtained using low-energy primary electrons, where fragments of different compositions are clearly visible (Figure 13a,b). By adjusting the imaging conditions, we were able to discriminate the two compositional phases constituting the precipitates in the steel matrix. At an applied energy of 1 keV of primary electrons, the precipitates composed of light elements appear bright in the SE1 image (blue arrows, Figure 13a) and dark in the corresponding BSE image (blue arrows, Figure 13b). Most probably, the grains of Y_2_O_3_ deliberately introduced into the mixture form the nucleation sites on which they crystallize. SEM observation shows nanometric precipitates distributed inside the grains and at the grain boundaries (Figure 13c). Careful EDS analysis performed on bigger precipitates (larger than 100 nm) revealed two different types of nanoparticles, namely free Y clusters containing Fe, Cr, and impurities, and Y-O-Ti rich clusters Y_2_O_3_ nanopowders initiate the growth of Fe-O-Cr precipitates. These results were confirmed by SEM micrograph analysis in composite contrast mode under different imaging conditions [8]. Numerous studies have been devoted to the analysis of nanooxides and other nanoprecipitates formed during both mechanical alloying and consolidation processes. Based on collected data obtained with advanced techniques such as transmission electron microscopy (TEM), atom probe tomography (APT), etc., it is now generally accepted that smaller nanoxides found in nanostructured ferritic materials are the pyrochlore phase of face-centered cubic Y_2_Ti_2_O_7_, while the larger ones are Y_2_TiO_5_ [31]. A high number density of well-dispersed oxides in the microstructure is crucial not only for improving mechanical properties and ensuring exceptional thermal stability but also for enhancing the radiation resistance of ODS materials. Due to the high interfacial area between the nanooxide–ferrite matrix and its ability to trap and manage helium atoms, the phenomenon of pore swelling is significantly limited.

The analysis confirmed a homogeneous submicron structure. Numerous submicron precipitates are visible, located mainly at the grain boundaries; precipitates of the order of 20–50 nm are present in the grain regions. EDS analysis shows that these precipitates are of metal carbide/oxide nature. In the SEM image, in channeling contrast, poorly formed grains with the size of 30 microns are visible.

**Figure 14:**
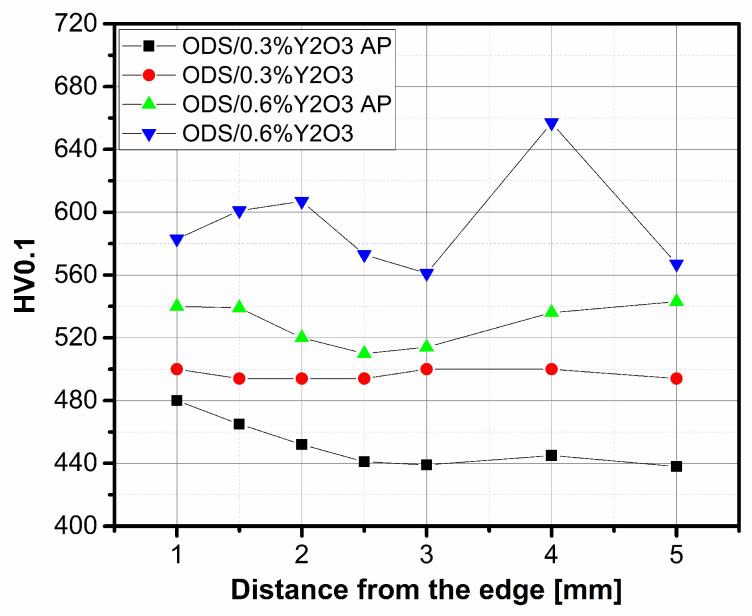
Hardness HV0.1 of ODS material at the distance from the edge.

The hardness HV0.1 of ODS steel samples was measured at the distance from the edge, and the obtained results are shown in Figure 14. A higher hardness value can be observed for ODS with 0.6% Y_2_O_3_ content. The significantly higher hardness value for higher yttrium oxide content is due to the fact that the ceramic grains stack up at the grain boundaries and cause a strengthening of the structure, which becomes more resistant to point loads. The hardness (VH0.1) values obtained in the range of 600–640 are very satisfactory. In the literature [13], a composite of the same composition obtained by the HIP technique had a hardness (HV0.1) of 470.

**Figure 15:**
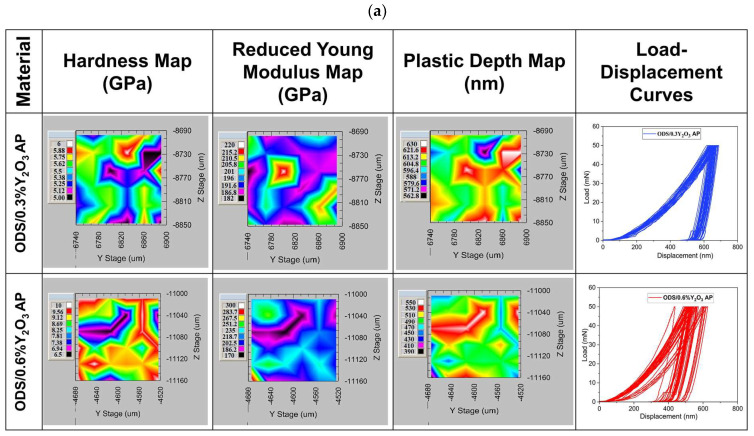

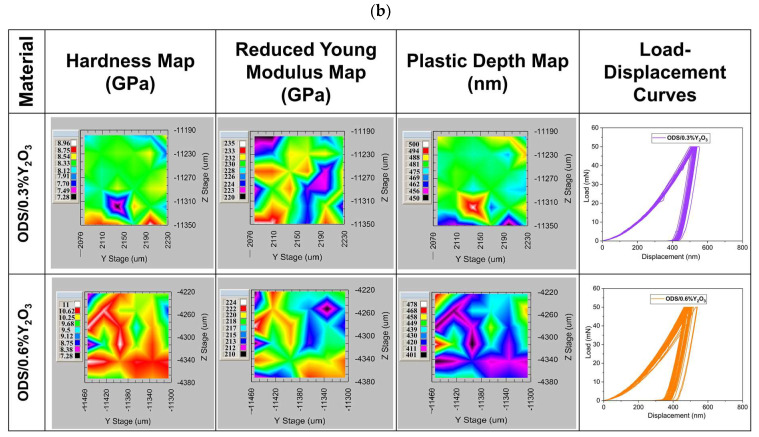
Maps of hardness, Young’s modulus, plastic depth, and load–displacement curves as a function of Y_2_O_3_ content in ODS steel obtained during 36 indentations; (**a**) for samples in which the powders were annealed (AP) and (**b**) for samples in which the powders were not annealed.

Maps of mechanical properties are shown in Figure 15. It can be seen that the hardness distribution varies depending on the addition of Y_2_O_3_. In ODS with 0.3% Y_2_O_3_, the hardness ranges from 5 to 6 GPa, while in 0.6% Y_2_O_3_, it ranges from 6.5 to 10 GPa. Such a hardness range may be related to different grain orientations, as seen in the SEM images in Figure 13. From the load–displacement curves, it can be seen that the indenter goes to similar depths in ODS 0.3% Y_2_O_3_, while in ODS 0.6% Y_2_O_3_, the depths obtained are slightly scattered. In addition, the plastic deformation ranges from 563 to 630 nm in ODS 0.3% Y_2_O_3_ and from 390 to 500 nm in ODS 0.6% Y_2_O_3_. This means that in ODS 0.6% Y_2_O_3_, we are dealing with harder grains, probably due to a higher amount of Y_2_O_3_ reinforcing phase, as seen in Figure 13 where Y_2_O_3_ is located both at grain boundaries and inside metallic grains. From the presented maps, a different range of reduced Young’s modulus can be observed, wherein ODS 0.3% Y_2_O_3_ varies between 182 to 220 GPa and in ODS 0.6% Y_2_O_3_ between 170 to 300 GPa. Higher Young’s modulus values in ODS 0.6% Y_2_O_3_ can be attributed to the superior stiffness of this material.

**Figure 16:**
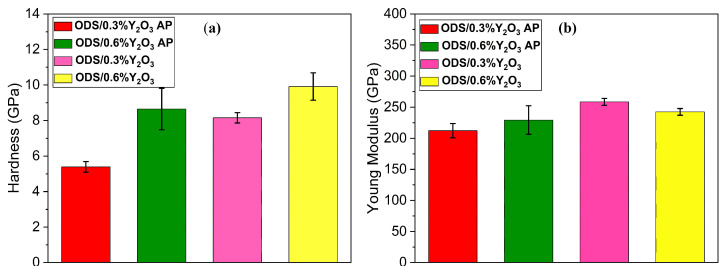
(**a**) An average hardness value of ODS with 0.3% Y_2_O_3_ and 0.6% Y_2_O_3_, (**b**) an average Young’s modulus value of ODS with 0.3% Y_2_O_3_ and 0.6% Y_2_O_3_ obtained during 36 indentations.

The average hardness and Young’s modulus values were then estimated and plotted in Figure 16. It is clear that the hardness of ODS 0.6% Y_2_O_3_ is about 37% higher than that of ODS 0.3% Y_2_O_3_. Interestingly, the calculated Young’s modulus is slightly higher in ODS 0.6% Y_2_O_3_, but the difference compared to ODS 0.3% Y_2_O_3_ is not as significant as in the case of hardness. Therefore, when analyzing and interpreting the properties of the obtained sinters, it is necessary to take into account not only the effect of different Y_2_O_3_ content but also the separation after the MA + SPS production process. In addition to the existing bcc Fe phase, the fcc Cr phase (as confirmed by X-ray and Mössbauer spectra), as well as carbides and oxides contribute to these structural changes, which determine, among other things, the mechanical properties of the resulting sinters.

## 4. Conclusions

ODS alloys seem to be very promising for future applications in Generation IV fission and fusion reactors. Our study showed successful preparation of Fe-Cr-W-Ti-Y_2_O_3_ ODS alloys using a combination of two processes: mechanical alloying and spark plasma sintering. XRD analysis revealed the presence of the basic bcc phase of α-Fe and a separate crystalline tungsten phase after the mechanical alloying process. At the same time, a significant decrease in the size of crystallites from 38 nm to about 14 nm was noticed after the mechanical alloying process. Mössbauer spectroscopy confirmed the formation of an α-Fe-based solid solution, and the process of annealing the powder under a nitrogen atmosphere leds to local atomic rearrangements in the solid solution. The observed subtle changes in the structure of ODS steel caused by the powder heat treatment process at 600 °C had a significant impact on the properties of the sinters obtained by the SPS technique. Based on the study, the following conclusions can be drawn:

⮚ The SPS consolidation process (1075 °C, 10 min, 10 °C/min, 50 MPa) allowed for good material densification (relative density over 98%), with a limited amount of pores and a lack of visible microcracks and structure discontinuities.
⮚ Microstructural investigations revealed two different types of uniformly distributed nanoparticles, namely free Y clusters containing Fe, Cr, and impurities, and clusters rich in Y-O-Ti, located at both grain boundaries and inside metallic grains,
⮚ The annealing process of powders after mechanical alloying resulted in the increase of the density of sinters in comparison to materials from non-annealed powders, which beneficially influenced the hardness, Young’s modulus, and thermal conductivity of ODS materials.

In summary, it can be concluded that it is possible to design the structure and properties of sintered materials already at the stage of preparing of powder mixtures by modifying the material composition or conducting thermal treatment of powders mixtures. This can result in the optimization of the materials’ microstructure in terms of their potential functionality. Our future plans will focus on modifying the chemical composition of ODS steel using alloying additives, which is in line with the general trend observed in the literature.

## Figures and Tables

**Table 1:** Hyperfine parameters obtained from fitting the Mössbauer spectra of ODS samples: hyperfine field (B_hf_), isomer shift (IS), and relative spectral area (A), with typical experimental uncertainties for B_hf_—0.1 T, IS—0.01 mm/s, and A—1.0% and 0.1% for sextets and singlets, respectively.

Materials	Spectral Component	B_hf_ [T]	IS [mm/s]	A [%]
ODS + 0.3% Y_2_O_3_	Fe-Cr	33.7 30.6 26.7	0.01 0.00 −0.02	11.5 38.5 50.0
ODS + 0.3% Y_2_O_3_ AP	Fe-Cr Cr-rich	34.0 30.9 27.2 -	0.01 0.00 −0.02 −0.07	8.0 39.5 51.9 0.6
ODS + 0.6% Y_2_O_3_	Fe-Cr	33.6 30.6 26.8	0.01 0.00 −0.02	17.7 37.4 44.9
ODS + 0.6% Y_2_O_3_ AP	Fe-Cr Cr-rich	34.1 30.9 27.2 -	0.01 0.00 −0.02 −0.06	9.4 46.6 43.6 0.4

**Table 2:** Results of density, thermal conductivity, specific heat capacity (Cp), and coefficient of linear expansion (CTE) at 50 °C of ODS sinters.

Materials	Measured Density [g/cm^3^]	Relative Density [%]	Thermal Conductivity [W/mK] at 50 °C	Specific Heat [J/gK] at 50 °C	CTE [1/K × 10^−5^] at 50 °C
ODS + 0.3% Y_2_O_3_	7.71	98.9	17.3 ± 0.6	0.55	1.04
ODS + 0.3% Y_2_O_3_ AP	7.78	99.7	17.6 ± 0.3	0.52	1.09
ODS + 0.6% Y_2_O_3_	7.67	98.4	17.4 ± 0.4	0.57	1.18
ODS + 0.6% Y_2_O_3_ AP	7.69	98.6	17.8 ± 0.2	0.54	1.17

## Data Availability

The data supporting the conclusions of this study can be obtained from the corresponding author upon reasonable request.
